# Devon and Exeter Hospital

**Published:** 1894-11-10

**Authors:** 


### THE INSTITUTIONAL WORKSHOP.

### WITHIN THE HOSPITALS.

### DEVON AND EXETER HOSPITAL.

By Our Special Commissioner.

In The Hospital of October 15th, 1892, pp. 46 and 47, we gave a description of the then condition of the Devon and Exeter Hospital, Exeter. We wrote at that time: "Go where he may, the eye of the visitor finds evidence of the urgent need which exists for an immediate redecoration, refitting, and refurnishing of the Devon and Exeter Hospital." We then proceeded to particularise the most glaring of the defects, and to urge the local press "to send some qualified person to go over the hospital, and see whether it was not precisely true that, owing to much wear and tear, the appearance of the wards and offices was far behind that of a modern Poor-Law infirmary."

Being at Exeter a fortnight ago, we thought it would be useful to make an inspection of the hospital, and ascertain if any and what changes had been introduced there during the last two years. As a result we can heartily congratulate the committee, its officers, and the local press, as well as the governors, upon the great improvement which is everywhere manifest throughout the building. The most noticeable, as it was the most gratifying, of all the changes made was the perfect cleanliness to be met with everywhere throughout the wards and corridors. The change in appearance and smartness was so marked as to make it difficult to realise that we were in the same building as that described in our columns two years ago. The nurses were alert, intelligent, and evidently took a pride in their work. The arrangements of the wards and several of the fittings had been improved or renovated, and the term used by a correspondent to describe the change is not too strong. The words were: "The Devon and Exeter Hospital has been so changed that its present condition, compared with that of two years ago, represents nothing less than a transformation scene, to the great credit of all concerned."

#### Then and Now.

One of the special features in the Devon and Exeter Hospital which we formerly described, was the separation wards for the treatment of severe cases after operation. In the old days, before Lister had inculcated intense cleanliness in the treatment of wounds, and when the wards wanted renovation and were often overcrowded, these separation wards were a great protection to the patients who had to undergo operations. At the present time the condition of the Devon and Exeter Hospital is so good, from a hygienic point of view, that we were assured it is the practice to move the operation cases into the general wards so that they may get a greater amount of air, and be placed under conditions entirely favourable to their speedy recovery. No higher evidence could be afforded of the important changes which have been made in the administration and structural arrangements of this old and famous hospital. Of course it is sometimes desirable still, for special reasons, to keep a patient in a separation ward for a few days immediately after the operation. But our congratulations are due to the medical staff that the general condition of the large wards is so good as to enable them to feel it to be better for the majority of operation cases to be taken back there rather than to be treated in the separation wards. We should like to see the operation theatre and its appurtenances smartened up and renewed. Possibly, in the structural alterations about to be made, to which we shall refer later, this point will receive the attention it deserves. Again, the practice at Exeter of devoting some of the beds to chronic cases, which we formerly commended, has been continued with the best results. Thus, two in-patients, who were in the hospital in 1892, are still in the wards under treatment. Seeing that the demand upon the beds is not greater than can be readily met, we are of opinion that it is of immense importance to the poor that a certain number of them in every county infirmary should be devoted, as at Exeter, to the reception of chronic cases. Another feature which gave us great satisfaction was the improvement which had taken place in the diet of the patients. The dinners were being served during the time of our visit, and we were much struck with the excellent quality of the food, and the appetising way in which it was served. We further welcome the addition of fresh green vegetables and puddings to the diets of those patients who, in the opinion of the medical staff, are able to eat them with advantage. In 1892 we noticed that the out-patients showed a tendency to decrease. This tendency has not been maintained however, for whereas the out-patients under treatment in 1891-92 amounted to 2,508, there were 2,797 out-patients under treatment in the year 1893-94, of whom 825 had previously been under treatment as in-patients.

#### Work yet to be Done.

Like most old buildings, the Devon and Exeter Hospital is now found to be ill-adapted for the needs of the increased staff which modern hospital requirements render necessary to the efficiency of an important institution like that at Exeter. The old buildings further require renovation and new sanitary fittings, which can best be provided by a reconstruction of the existing wards and the addition of a new wing for the reception of patients. The committee have caused plans to be prepared, which, when carried out, will make the central portion of the old buildings available for administration and the accommodation of the staff; the old wards will be immensely improved by the erection of turrets, in which the lavatories and bathrooms can be placed where they will be disconnected from the wards. We should counsel the committee, before finally settling the plans, to see the changes which have been introduced at the Sheffield General Infirmary and at the London Hospital, Whitechapel. At the latter hospital they will find specimens of the best slop-sinks, baths, and other fittings which are at present available for hospital purposes. We are confident, from the changes which the present committee have introduced since the date of our last visit, that it is their wish to secure the best of everything for their institution, and that is why we venture to suggest to them the propriety of basing their plan for reconstruction of the old wards and the selection of the new fittings upon the model of those which have recently been carried out at the London Hospital. In this connection it is a pleasure, as it is also a duty, to congratulate the House Surgeon, Mr. Henry Andrew, who was appointed in 1892; the Secretary, Mr. Albert E. Boyce; and the Lady Superintendent, Mrs. Brothers—the latter two of whom assumed office in April last—upon the noticeable changes to which we have referred, and the marked efficiency of the internal arrangements of the Devon and Exeter Hospital at the present time. Mrs. Brothers has introduced several excellent changes in the nursing system of the hospital, including extra leave, three years' training, and better remuneration. We should like to see the wages of the private staff nurses increased to at least £40 per annum, and those of the hospital staff nurses to £30 per annum, according to length of service. The amount of holiday leave is not stated, but we hope it is not less than three weeks each year in the case of all the nurses, and a month in the case of the sisters.

### Developments still Desirable.

We notice that the accounts are not yet kept on the uniform system. We hope, however, that Mr. Boyce, with the co-operation of the committee and auditors, will speedily set this matter right, because the adoption of the uniform system will enable the working of the Exeter Hospital to be compared on an identical basis with the best administered hospitals in the country. We further hope, as we said in our previous report, that the committee will communicate with Dr. Perry, of Guy's Hospital, and obtain a copy of the Guy's scheme of affiliation with the Royal National Pension Fund for Nurses. The adoption of a similar plan at Exeter would not only redound to the credit of the committee, but would at the same time ensure the maximum of efficiency and contentment amongst the members of the working staff, whose devotion to their duties entitles them to receive such a measure of encouragement as federation with the Royal National Pension Fund would enable the committee to offer. We cannot conclude this notice without thanking the editors of the local papers for the co-operation they extended to our efforts in 1892. We congratulate them upon the vast improvements which were everywhere manifest throughout the Devon and Exeter Hospital on the day of our visit. Exeter has an ably conducted local press, which takes a high place in provincial journalism, and Exeter citizens have a right to be proud of their local papers, which we have read with interest during the last twenty years, and which are conducted with a propriety and vigour that is worthy of all praise.

---

The compulsory notification of infectious diseases is now being insisted on by the Portuguese Government. But, in addition to the diseases which must be notified in England, the Portuguese authorities demand notification of plague, yellow fever, cerebro-spinal meningitis, whooping-cough, measles, influenza, puerperal fever, "green diarrhœa" of infants, purulent ophthalmia, and tuberculosis. For a nation which still believes in stringent quarantine this is fairly advanced.

